# Supplementary material for: Chronic and Gradual-Onset Injuries and Conditions in the Sport of Surfing: A Systematic Review
Source: Sports (Basel). 2021 Jan 29;9(2):23. doi: 10.3390/sports9020023 (PMC7911480; doi:10.3390/sports9020023)
Supplement: Supplementary file 1 [file sports-09-00023-s001.pdf]

Supplementary Material

***Table S1** – Modified AXIS Critical Appraisal Tool*

	Question	Yes = 1 or 2	No = 0	Not Applicable (Comment)
	**Introduction**			
1	Were the aims/objectives of the study clear?			
	**Methods**			
2	Was the study design appropriate for the stated aim(s)?			
3	Was the sample size justified?			
4	Was the target reference population clearly defined? (Is it clear who the research was about?)			
5	Was the sample frame taken from an appropriate population base so that it closely represented the target/reference population under investigation?			
6	Was the selection process likely to select subjects or participants that were representative of the target/reference population under investigation?			
7	Were measures undertaken to address and categorise non-responders?			
8	Were the risk factor and outcome variables measured appropriate for the aims of the study?			
9	Were the risk factor and outcome variables measured correctly using instruments/measurements that has been trialled, piloted or published previously?			
10	Is it clear what was used to determine statistical significance and/or precision estimate? (e.g. p-values, confidence intervals)			
11	Were the methods (including statistical methods) sufficiently described to enable them to be repeated?			
*12	Do the authors define their interpretation of injury? (e.g. chronic, gradual-onset or non-musculoskeletal conditions)			
13	Was an appropriate criteria for injury severity used?			
*14	Was injury data collected objectively? (Score = 2)<br>OR<br>Was injury data collected via self-reporting? (Score = 1)			
*15	If appropriate, was the mechanism of the injury recorded?			
	**Results**			
16	Were the basic data adequately described?			
*17	Are there no concerns about non-response bias?			
18	If appropriate, was information about non-responders described?			
19	Were the results internally consistent?			
20	Were the results presented for all the analyses described in the methods?			
	**Discussion**			
21	Were the authors' discussions and conclusions justified by the results?			
22	Were the limitations of the study discussed?			
	**Other**			
*23	Was there an absence of any funding sources or conflicts of interest that may affect the authors' interpretation of the results?			
24	Was ethical approval or consent of participants obtained?			
	**TOTAL SCORE =**			

Key: *Modified questions as per the Method section.

***Table S2** – Categorisation for Body Location of Musculoskeletal Injuries*

Category of Body Location	Inclusive of
Head/Face/Neck	Head/Face[10,24]; Neck[10,11,24]; Head/Neck[35]
Shoulder	Shoulder[10,11,24,29,35]
Upper Limb	Arm[10]; Forearm[35]; Elbow[11,24,29]; Wrist/Hand[24]
Torso	Ribs[11]; Ribs/Sternum[10]; Chest/Back[35]
Spine/Back	Back[11,29]; Thoracic[10,24]; Lower Back[10,23,24,35]
Hip/Groin	Hip/Groin[10,24]
Knee	Knee[10,24]
Lower Limb	Lower Limb[35]; Shin/Calf[24]; Leg/Ankle[10]; Ankle[24]
Other	Other[11,29]

***Table S3** – Categorisation for Mechanism of Musculoskeletal Injuries*

Category of Mechanism	Inclusive of
Paddling	Prolonged Paddling[24]; High Intensity Paddling[24]; Keeping Head Up While Paddling[24]; Overuse Paddling[4,5]; Paddling[10]
Manoeuvre	Turning Manoeuvres[24]; Landing Aerials[24]; Stress from Manoeuvre[4,5]
Overuse of Joint	Overuse of Joint[11]
Riding the Wave	Stand-Up Phase[24]; Pushing Down to Stand Up[24]; Tube Riding[24]; Trauma from Wave[24]; Riding Wave[10]
Prolonged Environmental Exposure	Prolonged Environmental Exposure[24]
Prolonged Positioning on Board	Prolonged Lying on Surfboard[24]; Prolonged Sitting on Surfboard[24]
Duck Diving	Duck Diving[24]
Certain Stances	Certain Stances[24]
Other	Miscellaneous[10]; Other[11]; Rib Inflammation[11]
Unknown	Unknown[10,24]

***Table S4** – Injury Epidemiology Specific to Injury Type, Location, Mechanism, & Severity*

Author (year)	Type of Injury	Body Region	Mechanism	Severity
Alexander et al.	Exostosis	Ear	-	Grade 0 (100% patency) = 95 (46%)<br>Grade 1 (66-99% patency) = 47 (23%)<br>Grade 2 (33-65% patency) = 34 (16%)<br>Grade 3 (<33% patency) = 31 (15%)
Altuna et al.	Exostosis	Ear	-	First group (<25%) = 16 surfers<br>Second group (25-50%) = 19 surfers<br>Third group (50-75%) = 8 surfers<br>Fourth group (75-100%) = 3 surfers
Attlmayr & Smith	Exostosis	Ear	-	No Obstruction = 76 (36.19%)<br>Mild (<30% obstruction) = 70 (33.33%)<br>Moderate (30-60% obstruction) = 38 (18.10%)<br>Severe (>60% obstruction) = 26 (12.38%)
Bazanella et al.	Chronic Low Back Pain	Lower Back	-	-
Deleyiannis et al.	Exostosis	Ear	-	Median scores (of both ears)<br><5yrs group = 7.5 obstruction<br>6-15yrs group = 63.0 obstruction<br>>15yrs group = 93.0 obstruction
Furness et al.	Chronic Injuries of;<br>Joint origin (43.5%)<br>Muscular origin (23.6%)<br>Nerve origin (4.6%)<br>Skin (0.5%)<br>Bone origin (3.9%)<br>Unspecified (16.2%)<br>Non-musculoskeletal origin (7.7%) - auditory exostosis and otitis externa, and eye injuries including pterygium and abscess	Head/Face = 103<br>Neck = 121<br>Shoulder = 254<br>Elbow = 39<br>Wrist/Hand = 22<br>Thoracic = 55<br>Lower Back = 314<br>Hip/Groin = 70<br>Knee = 160<br>Shin/Calf = 10<br>Ankle = 66	Prolonged paddling = 260 (21.1%) *<br>Turning manoeuvres = 182 (14.8%) **<br>Unknown = 142 (11.5%)<br>High-intensity paddling = 110 (8.9%) *<br>Stand-up phase = 76 (6.2%) **<br>Pushing down to stand up = 73 (5.9%) **<br>Keeping head up while paddling = 70 (5.7%) *<br>Prolonged environmental exposure = 68 (5.6%)<br>Prolonged lying on surfboard = 68 (5.6%)<br>Duck diving = 59 (4.8%)<br>Landing aerials = 31 (2.5%) **<br>Prolonged sitting on board = 29 (2.4%)<br>Tube riding = 24 (1.9%) **<br>Certain stances = 21 (1.7%) **<br>Trauma from wave = 18 (1.5%) **	-
Inada et al.	Chronic injuries or conditions of joint origin (100%)	Lower Back (31%)<br>Shoulder (27%)<br>Lower Limbs (16%)	-	-

		Head & Neck (15%)<br>Chest & Back (6%)<br>Forearm (5%)		
Kroon et al.	Exostosis	Ear	-	Normal (100% patency) = 125 (62%)<br>Mild (99-66% patency) = 53 (26%)<br>Moderate severe (<66% patency) = 24 (12%)
Lennon et al.	Exostosis	Ear	-	-
Lin et al.	Pterygium	Eye	-	Grade T1 (64.7%)<br>Occasional = 4<br>Recreational = 2<br>Enthusiast = 5<br>Grade T2 (17.6%)<br>Occasional = 0<br>Recreational = 0<br>Enthusiast = 3<br>Grade T3 (17.6%)<br>Occasional = 1<br>Recreational = 1<br>Enthusiast = 1
Lowdon et al.	Sprain, Strain	-	Overuse paddling = 26 (22%)<br>Stress from manoeuvre = 46 (39%)	-
Lowdon et al.	Sprain &/or Strain	-	Overuse paddling = 20 (29%)<br>Stress from manoeuvre = 29 (42%)	-
Nakanishi et al.	Exostosis	Ear	-	Grade 0 (no obstruction) = 150 (40.3%)<br>Grade 1 (<33% obstructed) = 118 (31.6%)<br>Grade 2 (33-66% obstruction) = 71 (19%)<br>Grade 3 (>66% obstruction) = 34 (9.1%)
Nathanson et al.	Overuse Syndromes (62%)<br>Environmental Exposure (18%)<br>Infection (15%)<br>Other (5%)	Shoulder (86)<br>Back (76)<br>Neck (43)<br>Knee (43)<br>Elbow (24)<br>Rib (14)<br>Other MSK (10)<br>Exostosis of ear (67)<br>Pterygium (19)<br>Otitis (33)<br>Cellulitis (29)<br>Sinusitis (10)	Overuse shoulder = 18%<br>Overuse back = 16%<br>Overuse neck = 9%<br>Overuse knee = 9%<br>Overuse elbow = 5%	-

		Other (23)		
Remnant et al.	Gradual-onset surfing-related injuries in the preceding 12 months	Shoulder (146)<br>Lower back (115)<br>Neck (104)<br>Knee (36)<br>Head and Face (35)<br>Upper Back (34)<br>Arm (24)<br>Ribs and Sternum (21)<br>Hip and Groin (21)<br>Leg and Ankle (13)	Prolonged paddling = 222 (28%) *<br>Stand up phase = 84 (10%) **<br>Head up whilst paddling = 70 (9%) *<br>High intensity paddling = 67 (8%) *<br>Preforming manoeuvre = 65 (8%) **<br>Prolonged lying on surfboard = 59 (7%)<br>Duck diving = 37 (5%)<br>Environmental exposure = 24 (3%)<br>Prolonged sitting = 19 (2%)<br>Moving neck during manoeuvre = 16 (2%) **<br>Landing aerials = 13 (2%) **<br>Wipe outs = 12 (2%) **<br>Tube riding = 8 (1%) **<br>Unknown or other = 105 (13%)	-
Simas et al.	Exostosis	Ear	-	Grade 0 (0% obstructed) = 9 (41.3%)<br>Grade 1 (1-33% obstructed) = 7 (30.4%)<br>Grade 2 (34-66% obstructed) = 3 (10.9%)<br>Grade 3 (67-100% obstructed) = 4 (17.4%)
Simas et al.	Exostosis	Ear	-	Bilateral = 293 (21.3%)<br>Unilateral = 104 (7.6%)
Taylor et al.	Chronic health problems (Exostosis, Pterygium, Sinusitis & MSK injuries)	Ear (67)<br>Eye (7)<br>Neck & Back (29)<br>Shoulder (15)<br>Knee (12)<br>Elbow (1)<br>Muscle/joint pain (10)<br>Stiffness (4)<br>Sinusitis (1)	-	-
Umeda et al.	Exostosis	Ear	-	Severe stenosis (0-5) unilaterally = 12<br>Severe stenosis (0-5) Bilaterally = 12<br>Normal to slight stenosis (6-10) = 37
Wong et al.	Exostosis	Ear	-	Normal (100% Patency) = 159 (26.5%)<br>Mild (99-66% Patency) = 239 (39.8%)<br>Moderate (65-33% Patency) = 104 (17.3%)<br>Severe (< 33% patency) = 98 (16.3%)

Key: *Paddling, ** Riding wave
